# Transactions of the Medical Association of the State of Missouri

**Published:** 1889-02

**Authors:** 


TRANSCTIONS OF THE MEDICAL ASSOCIATION OF THE STATE OF MISSOURI. Thirty-first Annual Session, held at Kansas City, April, 1888.

This volume is one of the largest that has come to hand this year. It contains 462 pages, printed by E. & E. Carreras, of St. Louis, and is simply excellent in typography and contents. The minutes take up but eleven pages of the book, while the other 451 pages are filled with splendid papers and discussions.

A. W. McAlester, M. D., of Columbia, is the President, and Drs. J. C. Mulhall, of St. Louis, and J. H. Duncan, of Kansas City, are the recording secretaries. Those who wish information on the "laws and appropriations of the Federal and State Governments in the interest of the public health," will find in this volume of tranactions a most splendid compilation by G. Hurt, M. D., of St. Louis.—B.

